# Antibacterial Activity of Two New Cassane Diterpenoids from *Caesaplinia pulcherrima* against *Bacillus cereus* by Damage to Cell Membrane

**DOI:** 10.3390/ijms24054917

**Published:** 2023-03-03

**Authors:** Zihan Zhang, Panpan Wang, Mengsong Chen, Lu Xie, Xiujuan Zhang, Yefan Shi, Wang Lu, Qiang Zhang, Chunhuan Li

**Affiliations:** Shaanxi Key Laboratory of Natural Products & Chemical Biology, Shaanxi Engineering Center of Bioresource Chemistry & Sustainable Utilization, College of Chemistry & Pharmacy, Northwest Agriculture and Forestry University, Yangling District, Xianyang 712100, China

**Keywords:** *Bacillus cereus*, cassane diterpenoids, antibacterial activity, membrane damage, growth curve

## Abstract

*Bacillus cereus*, a Gram-positive bacterium, is a food contaminant that threatens the health of thousands of people around the world. Because of the continuous emergence of drug-resistant strains, the development of new classes of bactericides from natural products is of high priority. In this study, two novel cassane diterpenoids (pulchins A and B) and three known ones (**3**–**5**) were elucidated from the medicinal plant *Caesaplinia pulcherrima* (L.) Sw. Pulchin A, with a rare “6/6/6/3” carbon skeleton, showed significant antibacterial activity against *B. cereus* and *Staphylococcus aureus*, with MIC values of 3.13 and 6.25 μM, respectively. Further investigation of its mechanism of antibacterial activity against *B. cereus* is also discussed in detail. The results revealed that the antibacterial activity of pulchin A against *B. cereus* may be caused by pulchin A interfering with bacterial cell membrane proteins, affecting membrane permeability and causing cell damage or death. Thus, pulchin A may have a potential application as an antibacterial agent in the food and agricultural industries.

## 1. Introduction

Food safety is a major public concern. A group of food-related health threats is caused by food pathogens and their toxins that result in disease and death worldwide [1]. *Bacillus cereus*, an example of a foodborne pathogen, is a Gram-positive, spore-forming rod bacterium. It is able to contaminate a variety of foods throughout the world due to the high resistance of its endospore to various stresses, which allows it to survive in unfavorable environments [2,3]. As the causative agent of many foodborne diseases, *B. cereus*, ingested as viable cells or spores, produces and secretes enterotoxins - which induces food poisoning, primarily manifested as diarrhea and emesis [4]. Moreover, the intestinal infection *B. cereus* also causes severe nongastrointestinal infections, including central nervous system infections, endocarditis, respiratory and urinary tract infections, and endophthalmitis in both immunocompromised and immunocompetent individuals [5]. Notably, *B. cereus* is an important health threat to the infant demographic. During a period of 7 years, 0.61% of bacteria-related acute diarrhea cases in infants were attributed to *B. cereus* in Kosovo [2]. Natural products feature an enormous diversity of complex chemical scaffolds that play a vital role in the drug discovery and development process [6,7]. The drug resistance of the pathogen represented by *B. cereus* seriously threatens the health of all human beings. Therefore, the need to develop new classes of bactericides with novel chemical scaffolds, binding targets, and antimicrobial mechanisms from natural products is urgent [8].

Cassane-type diterpenoids feature a backbone of three fused cyclohexane rings and a furan ring or an *α*, *β*-lactone ring and are the most distinctive specialized metabolites of medicinal plants from the genus *Caesalpinia*. Most of these cassane derivatives have a wide range of bioactivities, such as antiproliferative, anti-inflammatory, antimalarial, antiviral, and antibacterial effects [9]. In recent years, cassane compounds have attracted considerable interest from the medicinal chemistry community owing to their diverse structures and notable pharmacological profiles [10,11]. However, literature reporting on their antibacterial mechanism specifically against *B. cereus* is lacking. In this study, two new cassane diterpenoids (pulchins A and B), including one featuring a cyclopropane D ring (**1**), together with three known ones (**3**–**5**), were isolated from the medicinal plant *C. pulcherrima*. The antibacterial mechanism of pulchin A against *B. cereus* was further studied to fully understand its mode of action and the possible applications of pulchin A. Notably, this is the first time that the mode of action of pulchin A against *B. cereus* has been studied through physiological and biochemical experiments combined with electron microscopy.

## 2. Results and Discussion

### 2.1. Structural Elucidation of the Compounds

**Figure 1:**
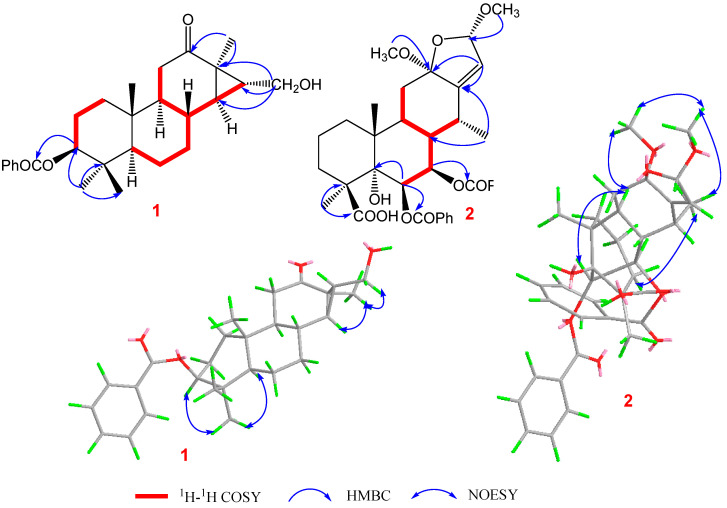
Key ^1^H-^1^H COSY, HMBC, and NOESY correlations of compounds **1** and **2**.

**Figure 2:**
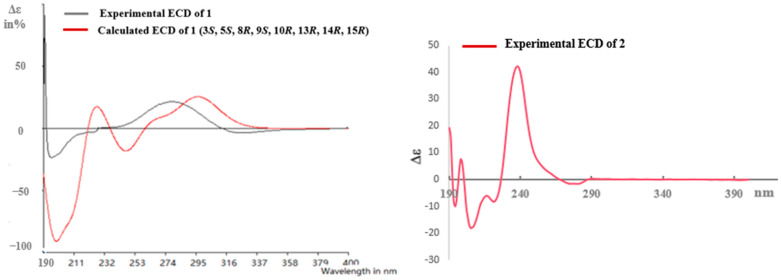
Calculated and experimental ECD for **1** and experimental ECD for **2**.

**Figure 3:**
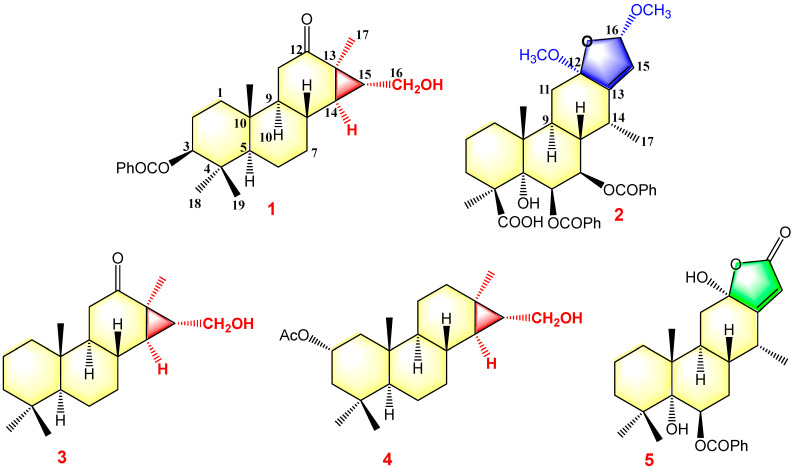
Structures of compounds **1**–**5**.

Pulchin A (**1**) was obtained in the form of white needles. Its molecular formula was assigned as C_27_H_36_O_4_ by the HREISMS spectrum in agreement with its ^1^H and ^13^C NMR data (Table 1). The ^1^H NMR spectral data exhibited four tertiary methyl groups at *δ*_H_ 0.91, 0.94, 1.05, and 1.18 (each 3H of singlet), and an oxymethylene at *δ*_H_ 3.48 (*dd*, *J* = 11.6, 7.7) and 3.74 (*dd*, *J* = 11.6, 5.8), together with an oxymethine at *δ*_H_ 4.71 (m). In the spectrum, a series of signals from the benzoyloxy group at values between *δ*_H_ 7.54 and 8.04 was also clearly observed. Other overlapping signals centered between 1.05 and 2.06 ppm are attributed to resonance from either methine or methylene signals. Seven carbon signals corresponding to the benzoyloxy group were not present in the ^13^C NMR spectrum. The 20 carbon resonances in the spectrum were further classified by DEPT experiments as four methyls (*δ*_C_ 14.4, 14.5, 17.1, and 28.5), six methylene groups with one oxygenated carbon (*δ*_C_ 61.7), six methines containing one oxygenated methine (*δ*_C_ 81.9), as well as four quaternary carbons including one keto carbonyl carbon (*δ*_C_ 210.6). The presence of a cyclopropane ring was deduced from the ^1^H-^1^H COSY cross-peaks of H-15 (*δ*_H_ 1.57, *dd*, *J* = 7.7, 5.8) with H-14 (*δ*_H_ 1.05, *m*) and H_2_-16 (*δ*_H_ 3.48, *dd*, *J* = 11.6, 7.7 and 3.74, *dd*, *J* = 11.6, 5.8); the HMBC correlations from H_2_-16 to C-13(*δ*_C_ 34.1), C-14 (*δ*_C_ 38.5), and C-15 (*δ*_C_ 37.9); and from Me-17 (*δ*_H_ 1.18, s) to C-12(*δ*_C_ 210.6), C-13, and C-14 (Figure 1). While the data implied a carbonyl group at C-12 and a hydroxyl group at C-16, a cyclopropane ring between the C-13, C-14, and C-15 formations was observed. Further analysis of the 1D and 2D NMR spectral data (Figures S1–S6) of compound **1** revealed similarities to those of pucherrin R (**3**) [12], which featured a cleistanthane backbone similar to compound **1**, but without a benzoyloxy group at C-3. This evidence was confirmed by the HMBC interactions of H-3 (*δ*_H_ 4.71, m) with C-2 (*δ*_C_ 24.4), C-4 (*δ*_C_ 38.8), Me-18 (*δ*_C_ 17.1), Me-19 (*δ*_C_ 28.5), and carbonyl carbon (*δ*_C_ 166.4). In the NOESY experiment, the correlations of H-3/Me-18 and Me-17/H_2_-16/Me-18 supported the *α* orientation of H-3, H_2_-16, Me-17, and Me-18 (Figure 1). The absolute configuration of **1** was established based on the comparison of experimental and calculated ECD data (Figure 2). Eventually, the absolute stereochemistry of this compound was defined as 3*S*, 5*S*, 8*R*, 9*S*, 10*R*, 13*R*, 14*R*, and 15*R*, as shown in Figure 3, owing to the agreement of the calculated ECD curve with the experimental one; this compound was named pulchin A. Notably, pulchin A with an exclusive cleistanthane backbone represents a rare class of cassane diterpenes.

Pulchin B (**2**) was obtained in the form of a white amorphous powder with the molecular formula C_36_H_42_O_10_, as determined by a combination of HREISMS (measured: *m*/*z* 657.2670 [M+Na]^+^, calculated: 657.2670) and NMR spectra, including ^1^H, ^13^C, and DEPT. The absorption bands at 3365 and 1716 cm^−1^ in the IR spectrum were ascribed to hydroxyl and ester carbonyl units, respectively. The ^1^H NMR spectrum revealed the presence of three methyls at *δ*_H_ 1.08 (3H, d, *J* = 7.3), 1.41 (3H, s), and 1.46 (3H, s); two methoxys at *δ*_H_ 3.13 (3H, s) and 3.41 (3H, s); and two oxygenated methines/olefinic protons at *δ*_H_ 5.90 (1H, dd, *J* = 11.3, 3.7) and 6.15 (1H, d, *J* = 3.7) and a pair of AB doublets at *δ*_H_ 5.33 and 5.63 (d, *J* = 1.1). Ten aromatic signals between *δ*_H_ 7.37 and 7.84 ppm and one singlet at *δ*_H_ 4.39 were also clearly observed in the ^1^H NMR spectrum. The combined analysis of ^13^C NMR (including its DEPT), HSQC, and HMBC of **2** showed 36 carbons, including two benzoyloxy groups (*δ*_C_ 166.6, 132.3, 2 × 130.4, 2 × 129.2, and 133.7; *δ*_C_ 166.0, 131.4, 2 × 130.3, 2 × 129.1, and 133.3); two methoxy groups (*δ*_C_ 50.4 and 55.9); one carboxyl carbon (*δ*_C_ 178.4); three methyls (*δ*_C_ 13.2, 17.5, and 25.0); four methines; seven methylenes, including three oxygen-occurring ones (*δ*_C_ 70.4, 72.3, and 106.6) and an olefinic carbon (*δ*_C_ 122.5); and five quaternary carbons involving two oxygenated ones (*δ*_C_ 78.8, and 110.5) and one olefinic carbon (*δ*_C_ 147.5). Further analysis of the HSQC data confirmed the assignment of all proton signals except for one at *δ*_H_ 4.39. This, in turn, confirmed that this signal corresponded to the free hydroxyl moiety. The above spectroscopic evidence suggests that compound **2** was also a highly oxygenated cassane diterpenoid.

A detailed analysis of the 1D and 2D NMR data (Figures S7–S14) of **2** revealed that its structure closely resembled that of caesalpulcherrin B, the first 2,5-dimethoxyfuranocassane diterpenoid, which was previously isolated from the aerial parts of *C. pulcherrima* [13]. The obvious difference between the two compounds was that an additional benzoyloxy group replaced the hydroxyl moiety located at C-7, as indicated by the HMBC correlations from H-7 (*δ*_H_ 5.90, dd, *J* = 11.3, 3.7) with C-6 (*δ*_C_ 70.4), C-8 (*δ*_C_ 40.8), C-14 (*δ*_C_ 31.9), and ester carbonyl carbon (C-1′’, *δ*_C_ 166.0); and the ^1^H-^1^H COSY interactions of H-7 with H-6 (*δ*_H_ 6.15, d, *J*= 3.7) and H-8 (*δ*_H_ 2.21, td, *J* = 11.3, 4.9) (Figure 1). Moreover, the long-range correlations between *δ*_H_ 1.41 (s, Me-18) and C-3 (*δ*_C_ 34.8), C-4 (*δ*_C_ 49.8), C-5 (*δ*_C_ 78.8), and carboxyl carbon (*δ*_C_ 178.4) revealed that Me-19 was carboxylated in compound **2**, conforming to its molecular formula. In the ROESY spectrum, the correlations of H-6/Me-17, H-6/Me-18, Me-17/H-7, Me-18/H-7, H-7/H-9, H-7/12-OMe, 12-OMe/Me-17, 12-OMe/16-OMe, 16-OMe/Me-17, and 16-OMe/H-9 clarified that H-6, H-7, H-9, Me-17, Me-18, 12-OMe, and 16-OMe were all in the same orientation (*α*) (Figure 2). Further analysis of the ROESY spectrum showed that the configurations of the remaining functional groups in **2** were the same as those in caesalpulcherrin B. The absolute configuration of **2** was then deduced as 4*S*, 5*S*, 6*S*, 7*S*, 8*s*, 9*S*, 10*R*, 12*R*, 14*R*, and 16*S*, as shown in Figure 3.

**Figure 4:**
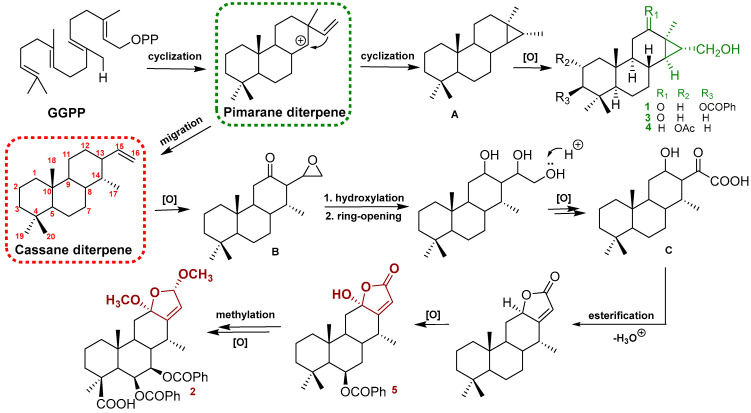
Plausible biogenetic pathways of compounds **1**–**5**.

The structures of the known cassane-type compounds (Figure 3) were elucidated by a combination of spectroscopic techniques and comparison with references. Compounds **3**–**5** were identified as pulcherrin R (**2**) [12], chaenocevhalol (**3**) [14], and pulcherritam F (**5**) [15], respectively. A plausible biosynthetic pathway for all isolated cassane derivatives is proposed (Figure 4). The precursor of cassane diterpene, pimarane diterpene, might be derived from geranylgeranyl pyrophosphate (GGPP) via intramolecular cyclization. Pimarane diterpene could be transformed into intermediate (A) via cyclization, which would subsequently undergo a series of oxidations to form compounds **1**, **3**, and **4**. Additionally, compounds **2** and **5**, the lactone-type cassane dierpenoids, could be generated from B by oxidation and esterification, as shown in Figure 4.

### 2.2. Antibacterial Activity of Compounds ***1***–***5*** In Vitro

As shown in Table 2, all five cassane derivatives were assessed for antibacterial effects against *B. cereus*, *Staphylococcus aureus*, and methicillin-resistant *Staphylococcus aureus* (MRSA). The results revealed that compounds **1**, **3**, and **4**, all of which feature a cleistanthane backbone, exhibited significant inhibitory activity against *B. cereus* with MICs of 3.13 μM, 6.25 μM, and 12.50 μM, respectively. Additionally, these three compounds exerted potential inhibitory effects against *S. aureus* according to their MICs (6.25 μM, 6.25 μM, and 12.50 μM); however, they displayed mild or no activity against MRSA. Compared with compounds **1**, **3**, and **4**, the other compounds **2** and **5** showed weaker inhibitory activity against both *B. cereus* and *S. aureus*. 

### 2.3. Inhibitory Effect of Compound ***1*** on the Growth of B. cereus and S. aureus

**Figure 5:**
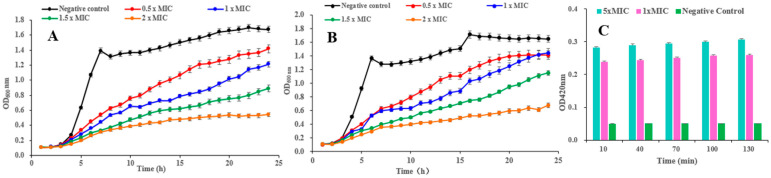
(**A**,**B**) Effects of compound **1** on the growth curves of *B. cereus* and *S. aureus*, respectively; (**C**) effect of **1** on the cell membrane permeability of *B. cereus*.

The growth curves of *B. cereus* and *S. aureus* treated with pulchin A (**1**) are shown in Figure 5A and Figure 5B, respectively. In the negative control groups, the OD_600nm_ increased at a faster rate in the first 8 h and remained stable until the end of the experiment. Compared with the control, each treatment with pulchin A at different concentrations (from 0.5 × MIC to 2 × MIC) delayed the exponential phase of cells and decreased the value of the growth curve of both *B. cereus* and *S. aureus*. In general, growth curve analysis revealed that compound **1** exerted remarkable inhibitory effects against these two bacteria, and the inhibitory effect was demonstrated to be dose- and time-dependent.

### 2.4. Effect of Compound ***1*** on the Cell Membrane Permeability of B. cereus 

*β*-galactosidase is widely found in various bacterial cells. Once the inner membrane of a bacterium is destroyed, *β*-galactosidase flows out into the culture medium, catalyzing a specific substrate, nitrophenyl *β*-D-galactopyranoside (ONPG), to yield o-introphenol (ONP), which has a strong absorption at 420 nm. Consequently, the content of extracellular *β*-galactosidase was measured to reflect the changes in the cell membrane permeability of *B. cereus*. As shown in Figure 5C, the extracellular bacteria treated with 1 × MIC and 5 × MIC of pulchin A (**1**) caused significantly higher membrane permeability than that in the control group. After being treated with 1 × MIC and 5 × MIC of **1** for 10 h, the OD_420 nm_ values were 5 and 5.6 times higher than that of the blank control, respectively. Over time, the OD values of *B. cereus* remained stable, implying that pulchin A may play an active role in destroying the inner membrane of cells during the first 10 h of contact with bacteria. Meanwhile, the OD values of *B. cereus* at 1 × MIC were similar to those at 5 × MIC, indicating that the inner membrane of cells may be destroyed at 1 × MIC. Consequently, the above results suggest that the cell membrane permeability increased after pulchin A treatment.

### 2.5. Scanning Electron Microscopy (SEM)

**Figure 6:**
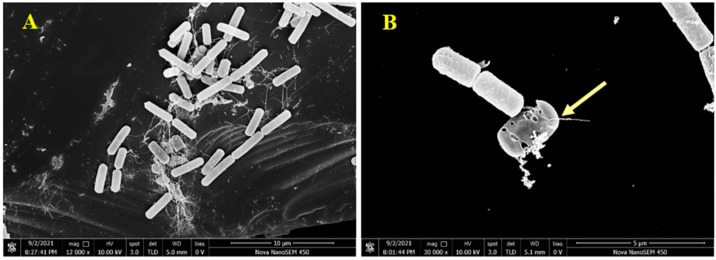
Scanning electron microscopy (SEM) images of *B. cereus*; (**A**,**B**) SEM images of control and treatment for *B. cereus* by **1**, respectively.

To visually analyze the action of pulchin A on the cell membrane, *B. cereus* morphology was observed using scanning electron microscopy (SEM). Compared with untreated *B. cereus* cells, the cellular morphology of *B. cereus* treated with compound **1** significantly changed. As shown in Figure 6A, the untreated *B. cereus* cells maintained a plump and smooth appearance, and their cell membranes were intact. In contrast, the yellow arrow reveals that the *B. cereus* cells treated with pulchin A (Figure 6B) showed an anomalous distorted shape and rupture, suggesting that the cell membrane was severely damaged. Cell membrane integrity is important for maintaining cell morphology and controlling normal cellular function. The SEM observations of the *B. cereus* cells revealed that pulchin A may disrupt the cell membrane by changing the cell permeability and cell integrity, which is likely to cause cell growth inhibition and death.

## 3. Materials and Methods

### 3.1. General Experimental Procedures

UV and ECD spectra were recorded using an Applied Photophysics Chirascan spectrometer (Applied Photophysics Ltd., London, U.K.). Optical rotations were measured on an MCP 300 photometer (Rudolph Research Aanlytical). The IR spectrum was obtained using a Bruker-Tensor-27 spectrometer (KBr pellets). The 1D and 2D NMR were obtained using a Bruker AMX-500spectrometer (Bruker, Rheinstetten, Germany) with TMS as an internal reference. HRESIMS spectra were obtained on an AB SCIEX Triple TOF 4600+ spectrometer (Thermo Fisher, Woburn, MA, USA). TLC analysis was carried out using silica gel 60 F254 and RP-18 F254S plates (Merck KGaA, Darmstadt, Germany). Silica gel (200–300 mesh, Marine Chemical Co., Ltd., Qingdao, China), RP-18 gel (20−45 μM, Fuji Silysia Chemical Ltd., Kasugai Aichi, Japan), and Sephadex LH-20 (40−70 μM, Amersham Pharmacia Biotech AB, Staffanstorp, Sweden) were used for column chromatography (CC). Semipreparative HPLC was achieved on an Agilent 1100 (Agilent T echnologies Inc., Santa Clara, CA, USA) series system using a 9.4 mm × 250 mm, 5 μM, YMC C18 column. 

### 3.2. Plant Material

*Caesalpinia pulcherrima* was collected in Yunnan Province, China, in July 2019, and identified by Dr. Ze-Huan Wang, State Key Laboratory of Phytochemistry and Plant Resources in West China, Kunming Institute of Botany, Chinese Academy of Sciences, Kunming, China. A voucher specimen (HITBC0021465) was deposited in the Herbarium of Xishuangbanan Tropical Botanical Garden, Chinese Academy of Sciences.

### 3.3. Extraction and Isolation

The dried stem (12.1 kg) was extracted with methanol three times. The organic solvents were evaporated under reduced pressure to yield a crude extract (562 g), which was suspended in water and partitioned with petroleum ether, EtOAc, and n-BuOH, successively. The EtOAc extract (193.8 g) was subjected to silica gel column chromatography eluted with CHCl_3_-Me_2_CO (1:0, 9:1, 8:2, 7:3, 1:1, and 0:1, *v*/*v*) to afford ten fractions (Frs. A–G) based on TLC analysis. 

Fraction A (28.6 g) was separated over a silica gel column chromatograph (CC), eluting with a PE- EtOAc gradient system (40:1–2:1, *v*/*v*) to yield six fractions (Frs. A1–A6). Hereafter, the subfraction A2 (1.8 g) was chromatographed with Sephadex LH-20 (CHCl_3_-MeOH, 1:1) to provide four subfractions A2.1-A2.4. A2.2 was subjected to semipreparative RP-HPLC with MeOH-H_2_O (88:12, *v*/*v*) to obtain compounds **1** (7.4 mg) and **4** (11.2 mg). A2.3 was repeated via ODS CC (MeOH-H_2_O, 85:15) and further purified with a MeOH-H_2_O system (78:22) using semipreparative HPLC to afford compound **5** (13.8 mg). Subfraction A2.4 (455 mg) was further applied to RP-C_18_ Me_2_CO/H_2_O (3:1–1:0, *v*/*v*) to obtain **2** (6.8 mg) and **3** (12.2 mg).

Pulchin A (**1**): white needles; [α]_D_^25^+91.49 (c = 0.4, MeOH); CD (MeOH) *λ*_max_ (Δε): 277 (+2.5), 196 (−2.7); UV (MeOH) λ_max_ (log *ε*) 190 (4.22) nm; IR (KBr) ν_max_ 3611, 2966, 2847, 1079, 1668, 1385, 1278,757cm^−1^; HR-ESI-MS m/*z* 447.2501 [M+Na]^+^ (calcd for C_27_H_36_O_4_Na, 447.2499).

Pulchin A (**2**): white amorphous powder; [α]_D_^25^+2.77 (c = 0.3, MeOH); CD (MeOH) *λ*_max_ (Δε): 238 (+42.3), 205 (−18.5); UV (MeOH) *λ*_max_ (log *ε*) 202 (2.53) nm; IR (KBr) ν_max_ 3365, 2928, 2384, 2311, 1716 cm^−1^; HR-ESI-MS m/*z* 657.2670 [M+Na]^+^ (calcd for C_36_H_42_O_10_Na, 657.2670).

### 3.4. Assay for Antibacterial Activity 

The minimal inhibitory concentration (MIC) was determined as an indicator of the antibacterial properties of the compounds [16,17]. Three test bacteria (*B. cereus*, *S. aureus*, and MRSA) were deposited at the College of Chemistry and Pharmacy, Northwest A&F University, China. The antibacterial activity assay was performed using the method of microdilution broth method in 96-well flat microtiter plates with minor modifications [11,16]. The MIC was defined as the lowest concentration of compounds capable of inhibiting the tested visible bacterial growth. Briefly, logarithmic-phase bacterial cells were adjusted to a concentration of 10^6^ CFU/mL. All compounds were dissolved in dimethyl sulfoxide (DMSO) to prepare the stock solution. The bacterial suspension (100 μL, 2 × 10^6^ CFU/mL) and 100 μL of compound solutions at concentrations ranging from 3.125 to 100 μM were gently added to the 96-well plate, successively. The absorbance at 600 nm was then examined with a microplate reader after incubation at 37 °C for 16 h. The MIC was defined as the lowest concentration of these compounds capable of inhibiting the tested visible bacterial growth. A system (DMSO) without samples was used as the negative control, while gentamicin was applied as the positive control. Each measurement consisted of three replicates. 

### 3.5. Measurement of the Bacterial Growth Curve

The influence of pulchin A (**1**) on the growth of bacteria was assessed using a modified version of the method developed by Lin [18]. First, the logarithmic-phase bacteria cells (*B. cereus* and *S. aureus*) were diluted to 10^6^ CFU/mL. Then, 100 μL of the above bacterial suspension and 100 μL of different concentrations (0.5 × MIC, 1 × MIC, and 2 × MIC) of sample solutions were incubated at 37 °C in a 96-well plate. Meanwhile, an equivalent volume of DMSO was added to the tube as the negative control. All tubes were cultured at 150 rpm at 37 °C. The absorbance values of 600 nm were then recorded every hour.

### 3.6. Measurement of Cell Membrane Permeability

When a bacterial membrane is damaged, small molecules as well as larger molecules, such as *β*-D-galactosidase, flow out [19]. Therefore, the integrity of the bacterial membrane can be evaluated by detecting the contents of *β*-D-galactosidase in solution over time. In brief, the logarithmic phase of *B. cereus* cells was collected by centrifugation at 4000 rpm for 5 min, washing twice with PBS (0.1M, pH7.0), and resuspending to 10^6^ CFU/mL. The bacterial suspension (10^6^ CFU/mL), *o*-nitrophenol-*β*-D-galactoside (ONPG), with the addition of two concentrations of pulchin A (0 × MIC, 1 × MIC, and 5 × MIC), were first incubated for 10, 40, 70, 100, and 130 min at 37 °C. An equivalent volume of LB medium liquid containing DMSO was used as the negative control. The UV absorption was then measured at 420 nm to determine the content change of *β*- galactosidase in solution over time.

### 3.7. Scanning Electron Microscopy (SEM)

SEM studies were conducted to observe the cellular morphological changes of *B. cereus* treated with pulchin A based on the method of Lin et al., with minor modifications [18,20]. Logarithmic-phase *B. cereus* cells (approximately 2 × 10^6^ CFU/mL) were treated with pulchin A at levels of 0 × MIC and 1 × MIC at 37 °C for 10 h. Then, the bacteria were collected, washed with PBS (0.1M, pH7.0), and anchored with glutaraldehyde. Specimens were dehydrated in various concentrations of a water–alcohol solutions (10, 30, 50, 70, 80, 90, and 100%) for 10 min each, and then coated with gold–palladium under vacuum. A field-emission scanning electron microscope (Nova Nano SEM-450, FEI Instruments, Inc., Hillsboro, Oregon) was used for the microstructural valuation of *B. cereus*.

### 3.8. Statistical Analysis

All experiments were performed at least 3 times. Statistical comparisons were performed using the SPSS version 16.0 (IBM, Armonk, NY, USA). The data are expressed as the mean values ± standard deviation. A probability value of *p* < 0.05 was considered statistically significant.

## 4. Conclusions

Two novel cassane diterpenoids (pulchins A and B), as well as three known ones (**3**–**5**), were discovered from the stem of the medicinal plant *C. pulcherrima*. Currently, pulchin A belongs to a rare group of cassane diterpenes that feature a clesitanthane backbone. The antibacterial mechanism of pulchin A against *B. cereus* was investigated by measuring the growth curve, membrane permeability, and SEM analysis. Based on the results, the antimicrobial effect of pulchin A against *B. cereus* can likely be attributed to the action of pulchin A on the cell membrane permeability of *B. cereus*. By increasing cell membrane permeability, pulchin A inhibited growth and caused cell death of *B. cereus*. To the best of our knowledge, this is the first time that the mode of action of pulchin A against *B. cereus* has been elucidated through physiological and biochemical experiments combined with electron microscopy. This work provides a novel insight for studying the antimicrobial action of pulchin A against *B. cereus* and has potential applications as a potential alternative food preservative in the food industry.

## Figures and Tables

**Table 1:** ^1^H and ^13^C NMR spectroscopic data of compounds **1** and **2** (*δ*_H,_ *δ*_C_ [ppm], *J* [ Hz]).

Position	1 ^[a]^		2 ^[b]^	
	*δ*_H_	*δ*_C_	*δ*_H_	*δ*_C_
1	1.16 m,1.71 m	36.6 t	1.80 m, 1.90 m	34.6 t
2	1.16 m, 1.81 m	24.4 t	1.52 m, 2.30 m	20.0 t
3	4.71 m	81.9 d	1.65 m, 1.86 m	34.8 t
4		38.9 s		49.8 s
5	1.14 m	57.5 d		78.8 s
6	1.32 m, 2.07 m	37.0 t	6.15 d (3.6)	70.4 d
7	1.51 m,1.75 m	22.4 t	5.90 dd (11.3,3.7)	72.3 d
8	1.14 m	54.4 d	2.21 td (11.3,4.9)	40.8 d
9	1.77 m	37.4 d	2.53 td (12.5,3.0)	38.3 d
10		37.6 s		42.1 s
11	1.32 m, 2.12 m	35.8 t	1.33 m, 2.29 m	40.1 t
12		210.6 s		110.5 s
13		34.1 s		147.5 s
14	1.05 m	38.5 d	2.95 m	31.9 d
15	1.57 m	37.9 d	5.63 d (1.1)	122.5 d
16	3.48 dd (11.6,7.7), 3.74 dd (11.6,5.8)	61.7 t	5.33 d (1.1)	106.6 d
17	1.18 s	14.4 q	1.08 d (7.3)	13.2 q
18	1.05 s	17.1 q	1.41 s	25.0 q
19	0.94 s	28.5 q		178.4 s
20	0.91s	14.5 q	1.46 s	17.5 q
5-OH			4.39 s	
12-OCH_3_			3.41 s	55.9 q
16-OCH_3_			3.13 s	50.4 q
1’		166.4 s		166.6 s
2’		131.7 s		132.3 s
3’	8.04 d (6.4)	130.1 d	7.84 d (6.7)	130.4 d
4’	7.53 t (7.7)	129.4 d	7.47 t (7.7)	129.2 d
5’	7.65 t (7.4)	133.8 d	7.6 1overlap	133.7 d
6’	7.53 t (7.7)	129.4 d	7.47 t (7.7)	129.2 d
7’	8.04 d (6.4)	130.1 d	7.84 d (6.7)	130.4 d

^[a] 1^H (400 MHz) and ^13^C (100 MHz) NMR recorded in methanol-*d_4_*; ^[b] 1^H (400 MHz) and ^13^C (100 MHz) NMR recorded in acetone-*d_6_*. The other benzoyloxy moiety signals of **2**: *δ*_H_7.81 d (6.9), 7.37 t (7.8), 7.58 overlap, 7.37 t (7.8), 7.81 d (6.9); *δ*_C_ 166.0 s, 131.4 s, 130.3 d, 129.1 d, 133.3 d, 130.3 d (7-OCOPh).

**Table 2:** MIC values of compounds **1**–**5** against test bacteria.

Compounds	MICs (μM) ^[a]^			Compounds	MICs (μM)		
	*B. cereus*	*S. aureus*	MRSA		*B. cereus*	*S. aureus*	MRSA
**1**	3.13	6.25	50.00	**4**	12.50	12.50	>100
**2**	25.00	75.00	>100	**5**	12.50	25.00	75.00
**3**	6.25	6.25	50.00	**Gentamicin ^[b]^**	3.13	3.13	12.50

^[a]^ MICs (μM): The minimal inhibitory concentration. ^[b]^ Positive control of three test bacteria.

## Data Availability

Not applicable.

## Supplementary Materials

The supporting information can be downloaded at: https://www.mdpi.com/article/10.3390/ijms24054917/s1.
