# Supplementary material for: Antibacterial Activity of Two New Cassane Diterpenoids from Caesaplinia pulcherrima against Bacillus cereus by Damage to Cell Membrane
Source: Int J Mol Sci. 2023 Mar 3;24(5):4917. doi: 10.3390/ijms24054917 (PMC10003239; doi:10.3390/ijms24054917)
Supplement: Supplementary file 1 [file ijms-24-04917-s001.zip › ijms-2209727-supplementary.pdf]

Supporting information

Antibacterial Activity of Two New Cassane Diterpenoids from *Caesaplinia pulcherrima* against *Bacillus cereus* by Damage to Cell Membrane

Zihan Zhang, Panpan Wang, Mengsong Chen, Lu Xie, Xiujuan Zhang, Yefan Shi, Wang Lu, Qiang Zhang and Chunhuan Li *

Shaanxi Key Laboratory of Natural Products & Chemical Biology, Shaanxi Engineering Center of Bioresource Chemistry & Sustainable Utilization, College of Chemistry & Pharmacy, Northwest Agriculture and Forestry University, Yangling District, Xianyang 712100, China

* Correspondence: chunhuanli@nwsuaf.edu.cn

Figure S1. ^{1}H NMR spectrum of **1** in Methanol-d_4

Figure S2. ^{13}C NMR spectrum of **1** in Methanol-d_4

Figure S3. ^{1}H-^{1}H COSY spectrum of **1** in Methanol-d_4

Figure S4. HSQC spectrum of **1** in Methanol-d_4

Figure S5. HMBC spectrum of **1** in Methanol-d_4

Figure S6. NOESY spectrum of **1** in Methanol-d_4

Figure S7. ^{1}H NMR spectrum of **2** in Acetone-d_6

Figure S8. ^{13}C NMR spectrum of **2** in Acetone-d_6

Figure S9. ^{1}H-^{1}H COSY spectrum of **2** in Acetone-d_6

Figure S10. HSQC spectrum of **2** in Acetone-d_6

Figure S11. HMBC spectrum of **2** in Acetone-d_6

Figure S12. NOESY spectrum of **2** in Acetone-d_6

Figure S13. The HRESIMS spectrum of **1**.

Figure S14. The HRESIMS spectrum of **2**.

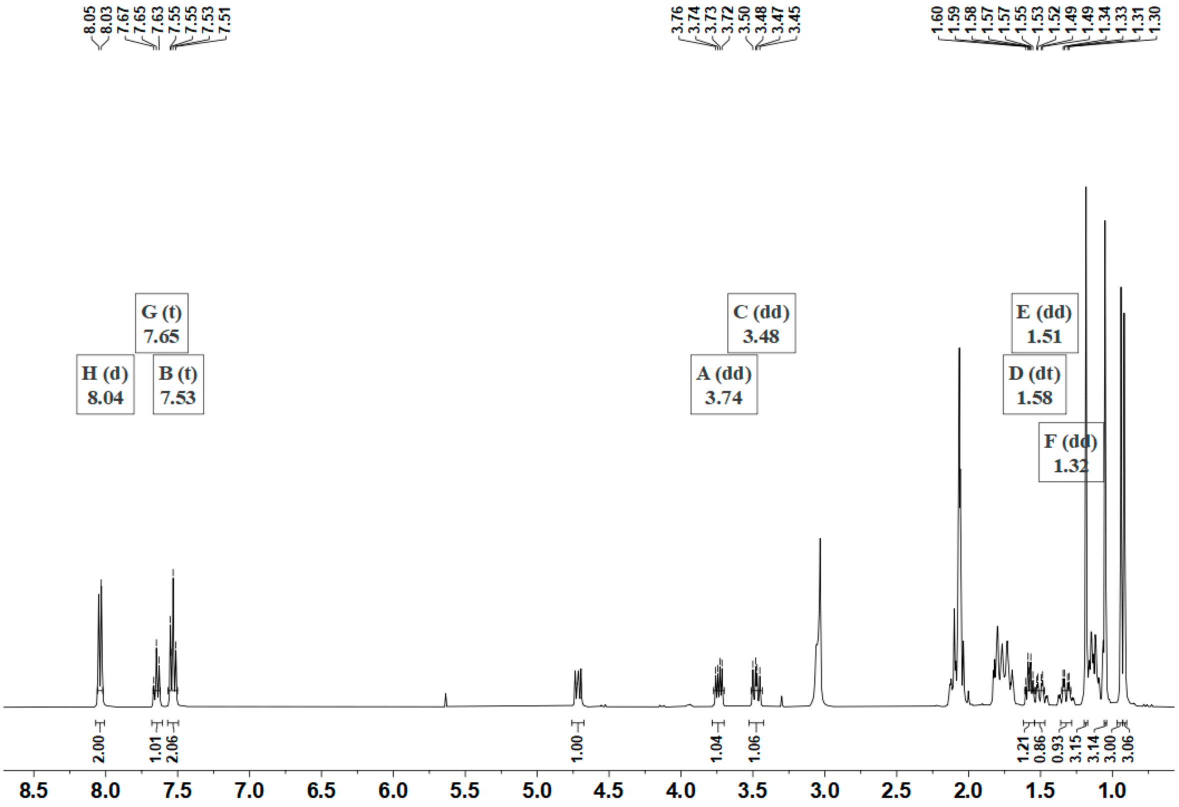

Figure S1. ^{1}H spectrum of **1** in Methanol-*d4*

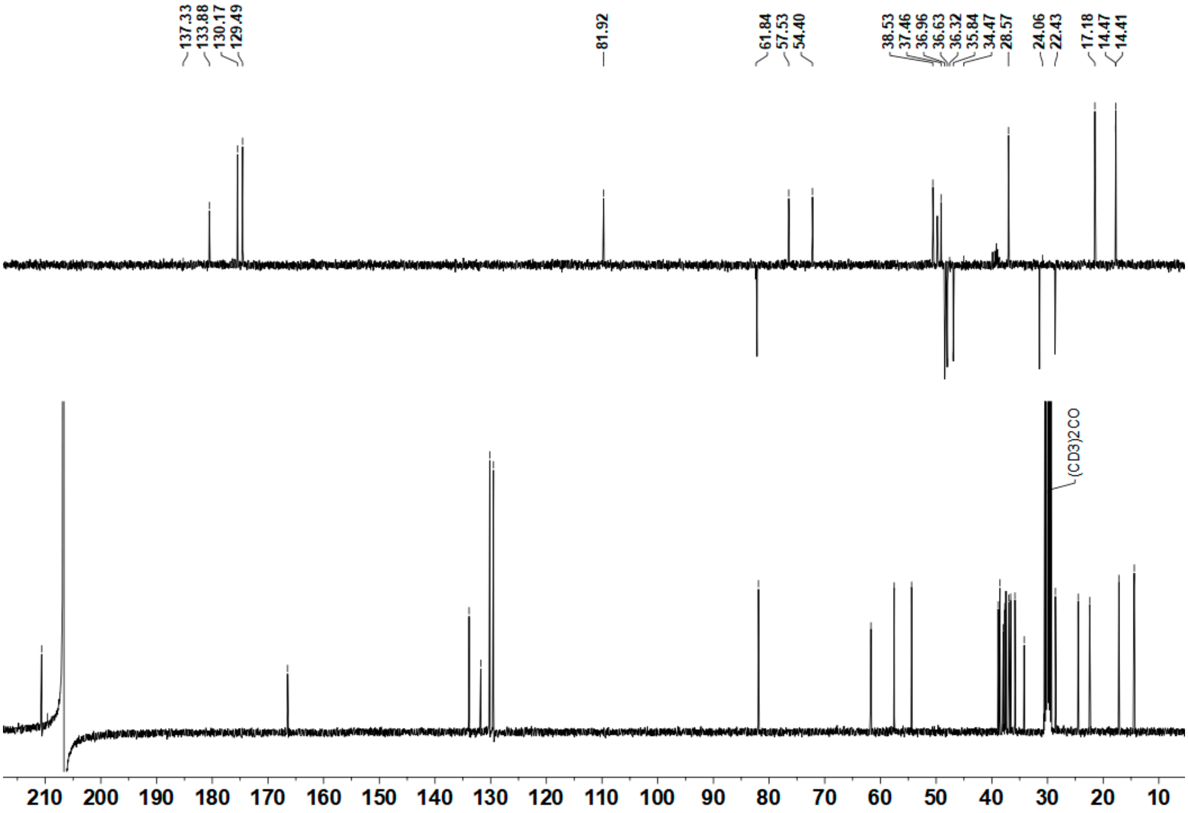

Figure S2. ^{13}C NMR spectrum of **1** in Methanol-*d4*

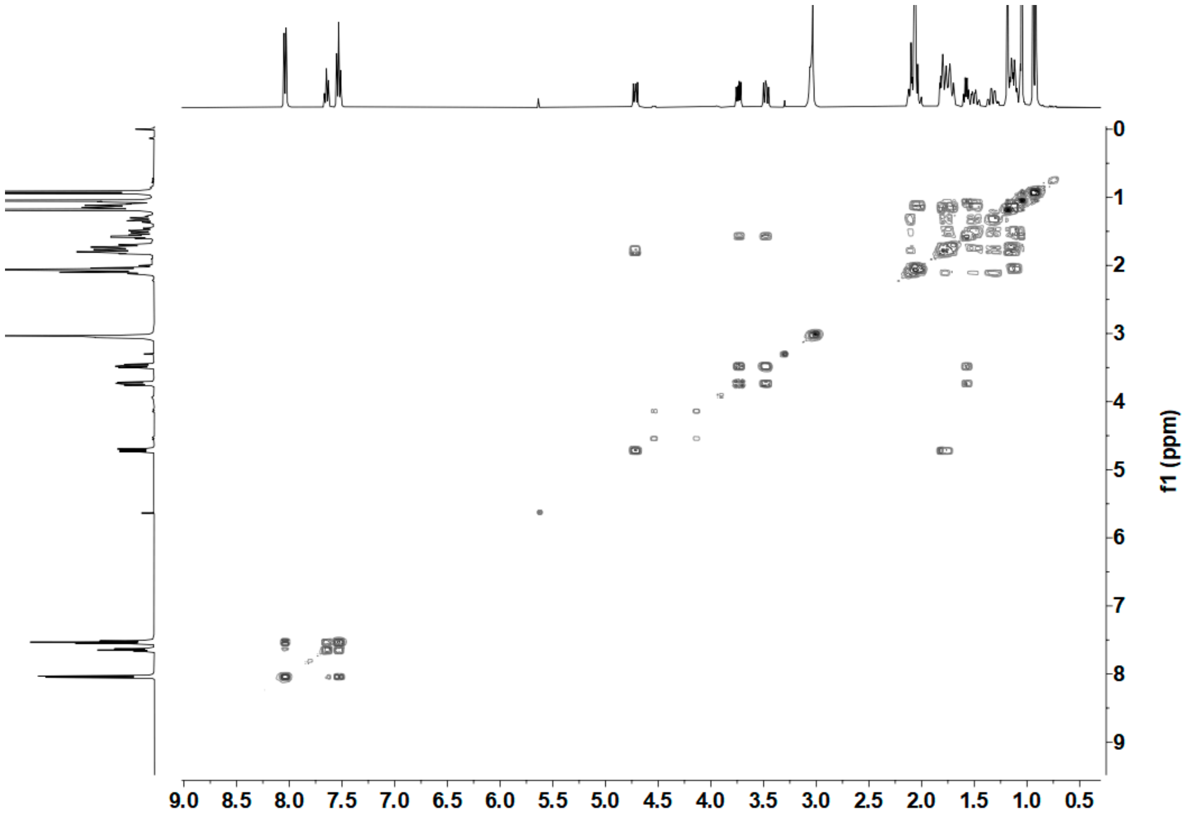

Figure S3. ^{1}H-^{1}H COSY spectrum of **1** in Methanol-d_4

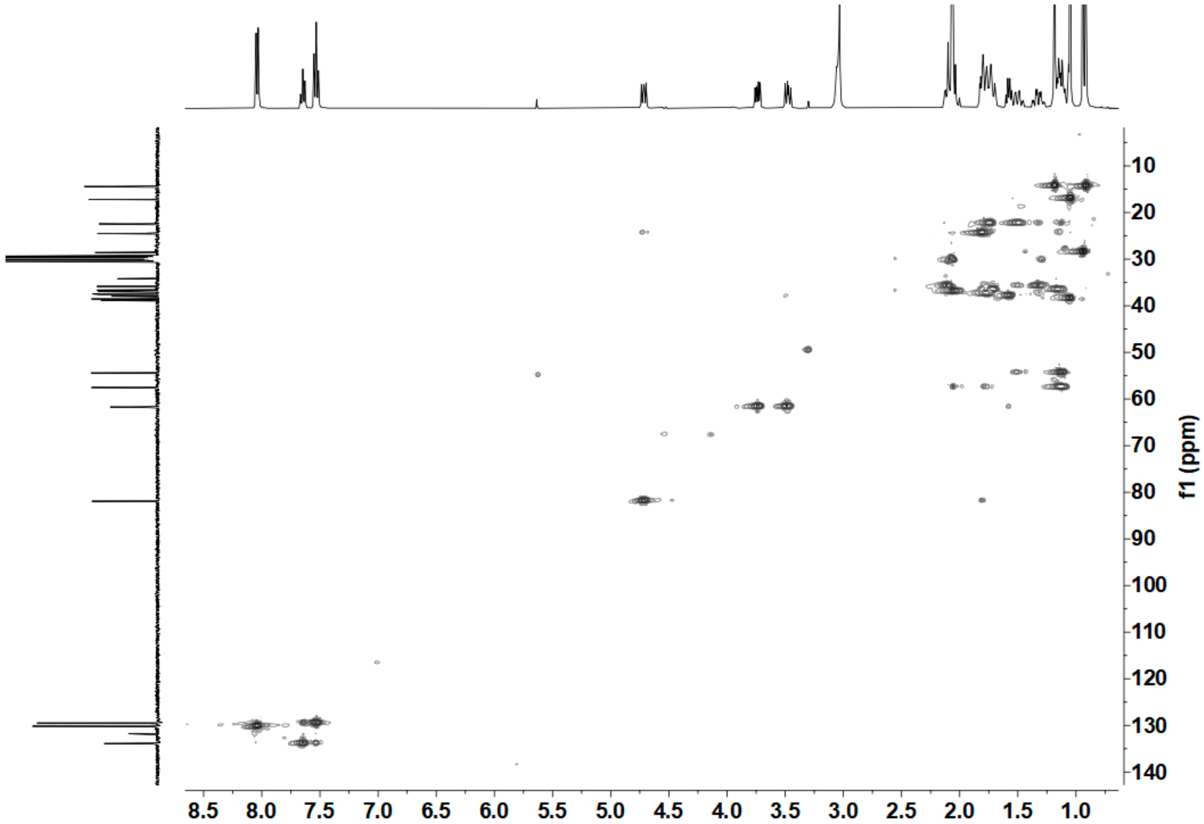

Figure S4. HSQC spectrum of **1** in Methanol-d_4

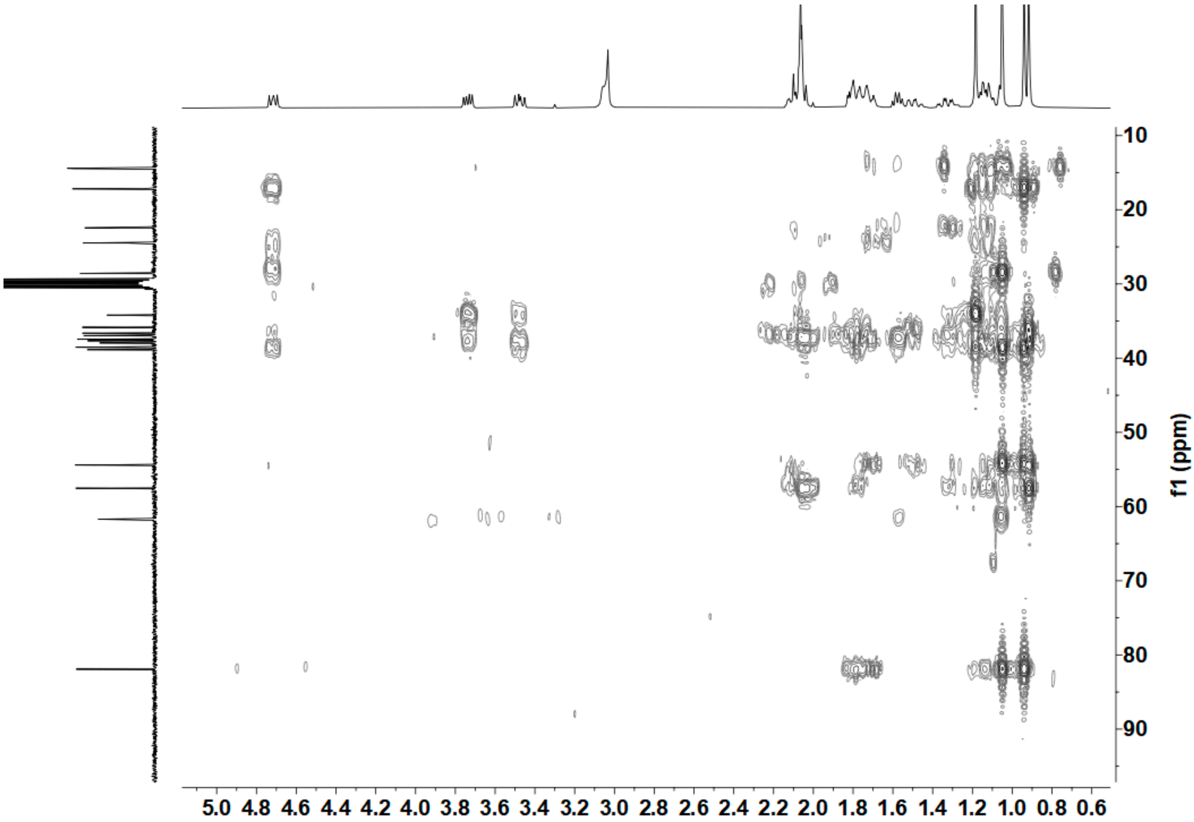

Figure S5. HMBC spectrum of **1** in Methanol-d_4

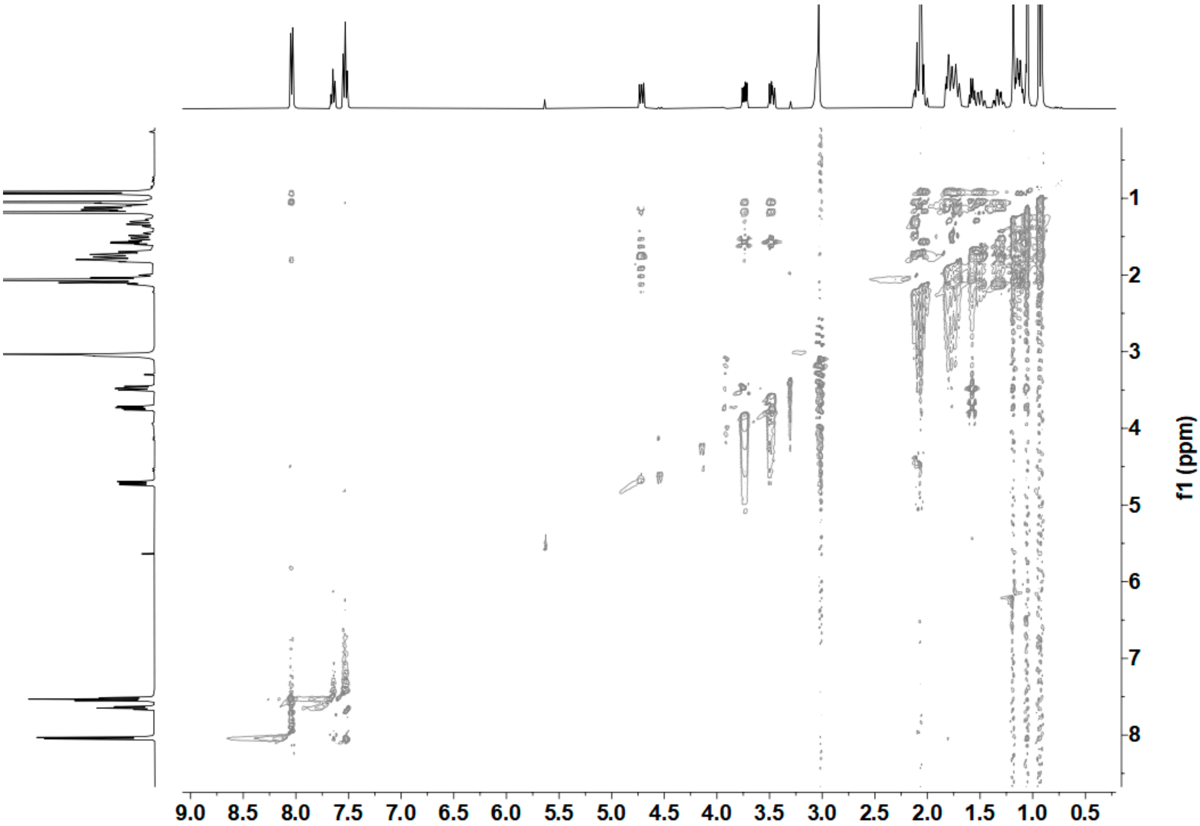

Figure S6. NOESY spectrum of **1** in Methanol-d_4

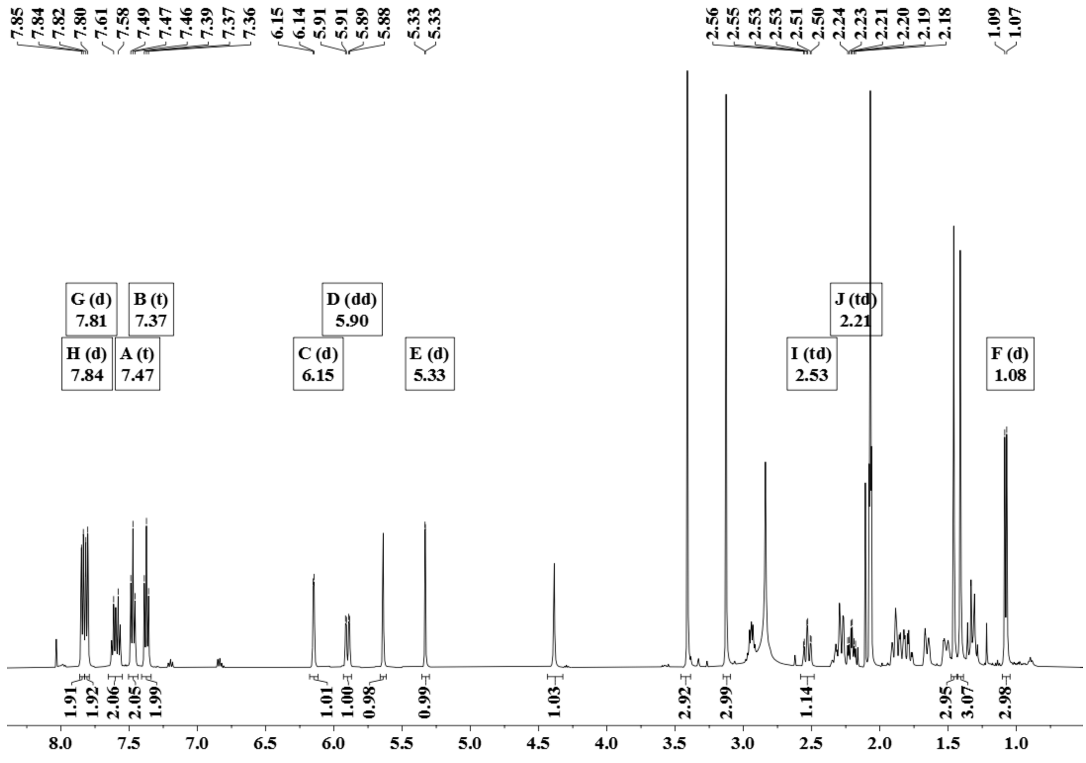

Figure S7. ^{1}H spectrum of **2** in Acetone-d_6

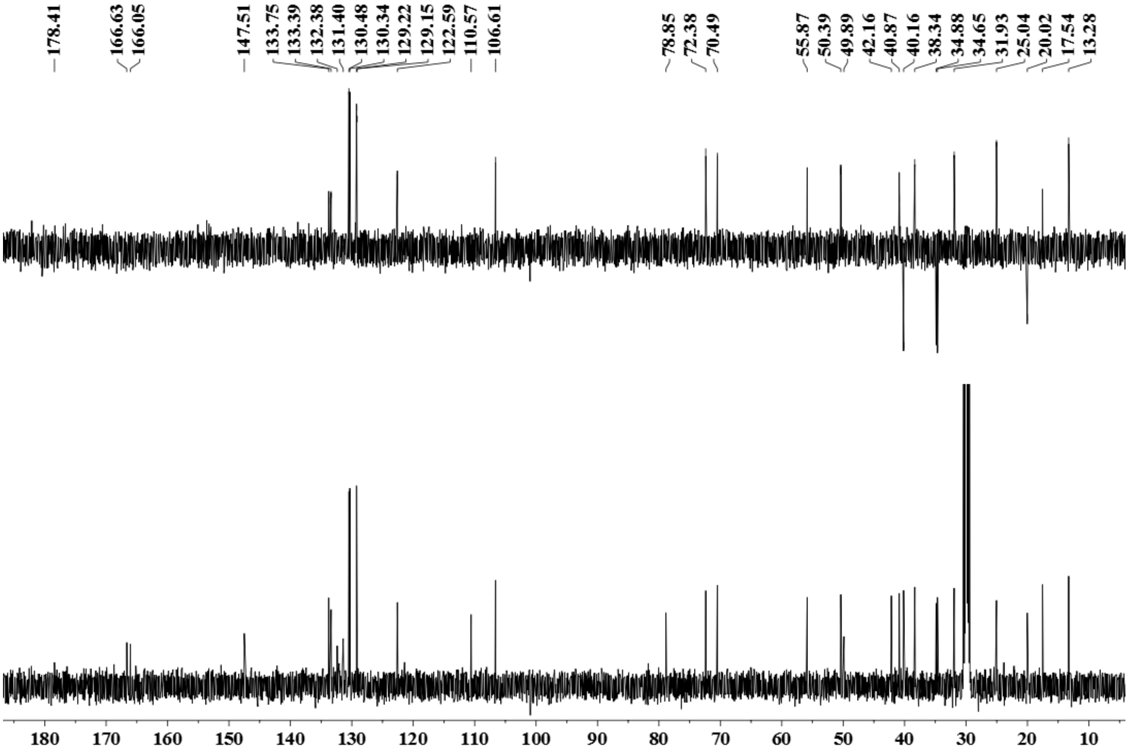

Figure S8. ^{13}C NMR spectrum of **2** in Acetone-d_6

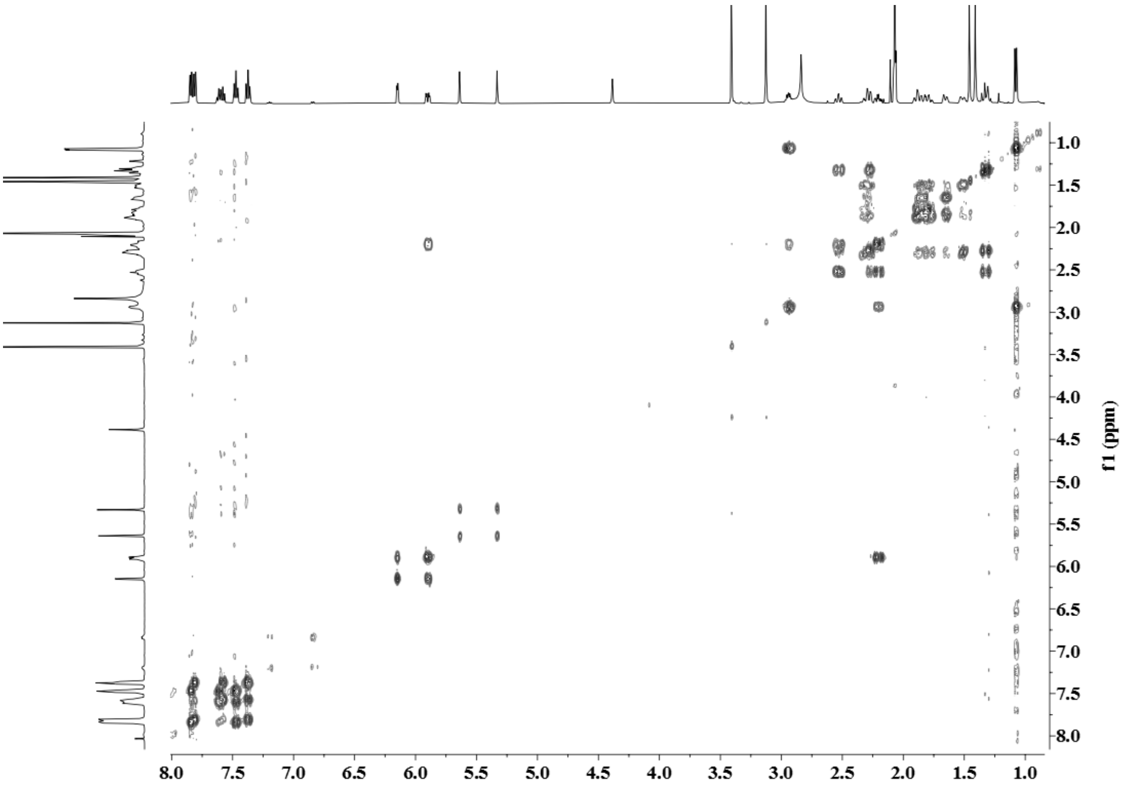

Figure S9. ^{1}H-^{1}H COSY spectrum of **2** in Acetone-*d6*

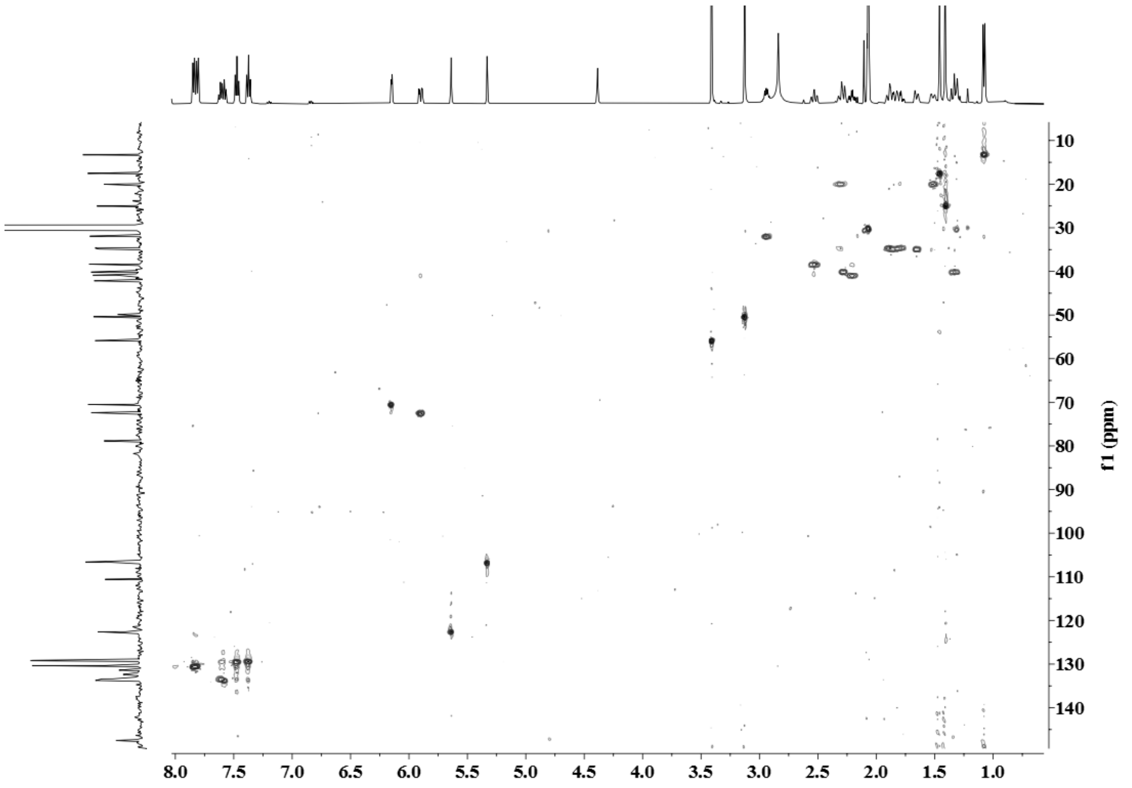

Figure S10. HSQC spectrum of **2** in Acetone-*d6*

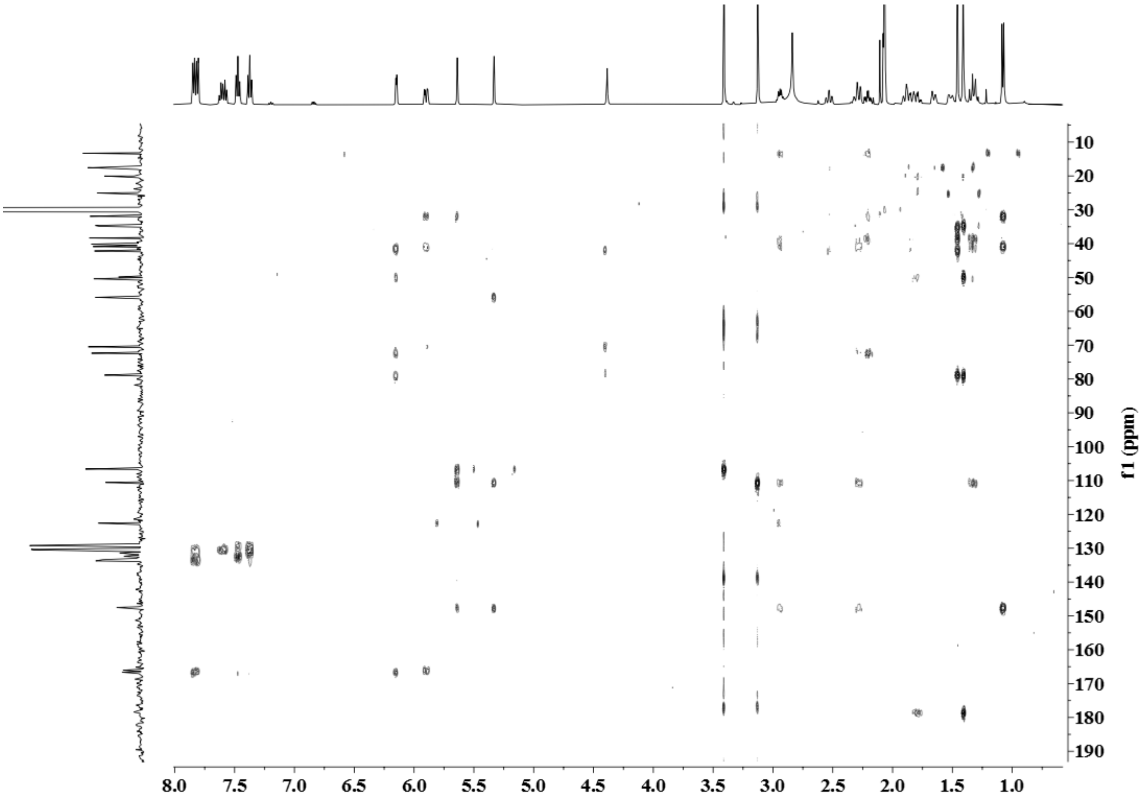

Figure S11. HMBC spectrum of **2** in Acetone-d_6

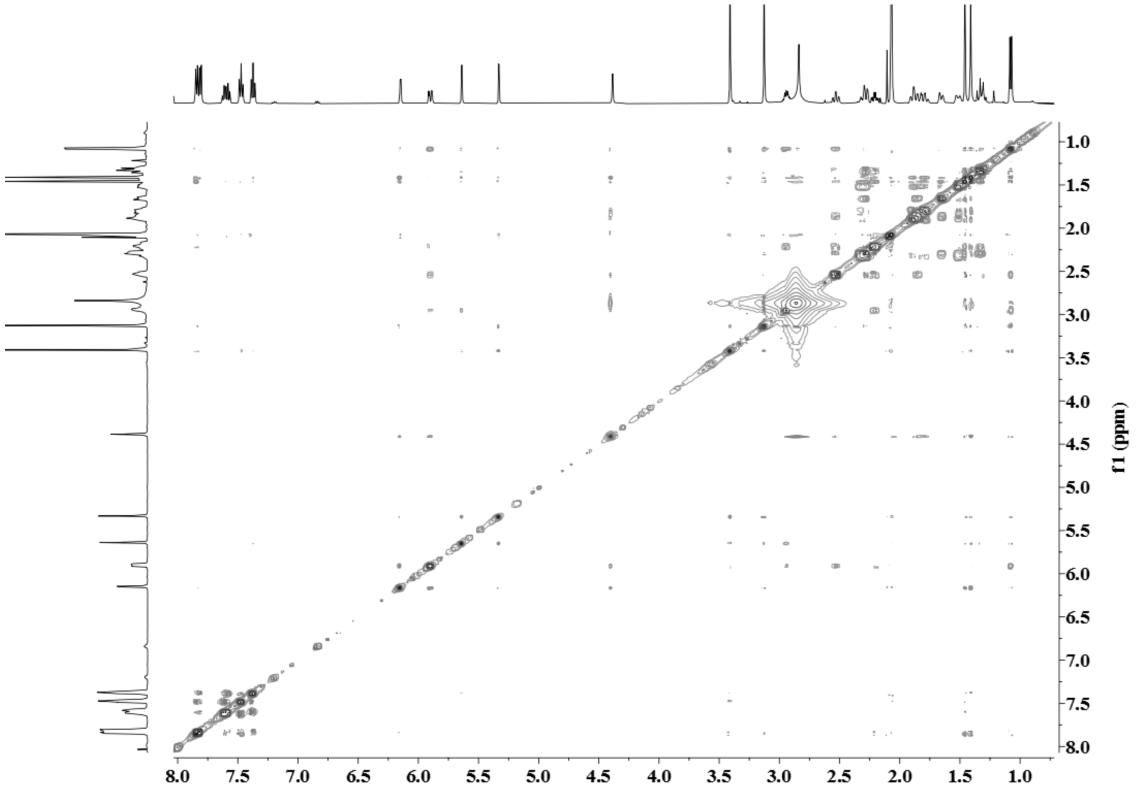

Figure S12. NOESY spectrum of **2** in Acetone-d_6

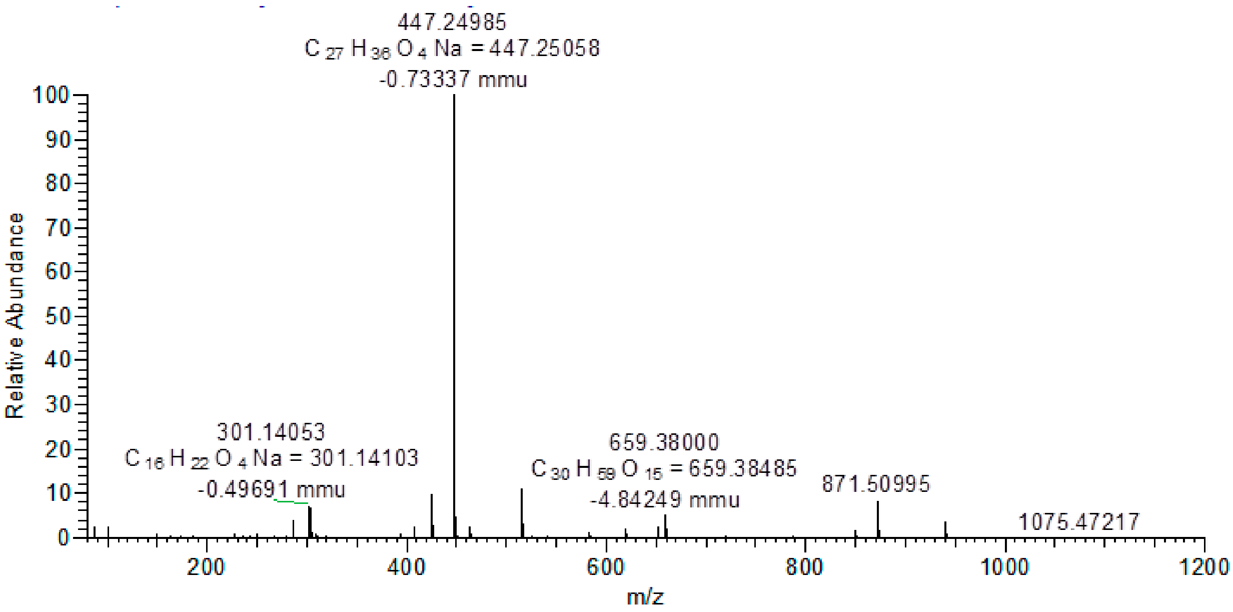

Figure S13. The HRESIMS spectrum of **1**.

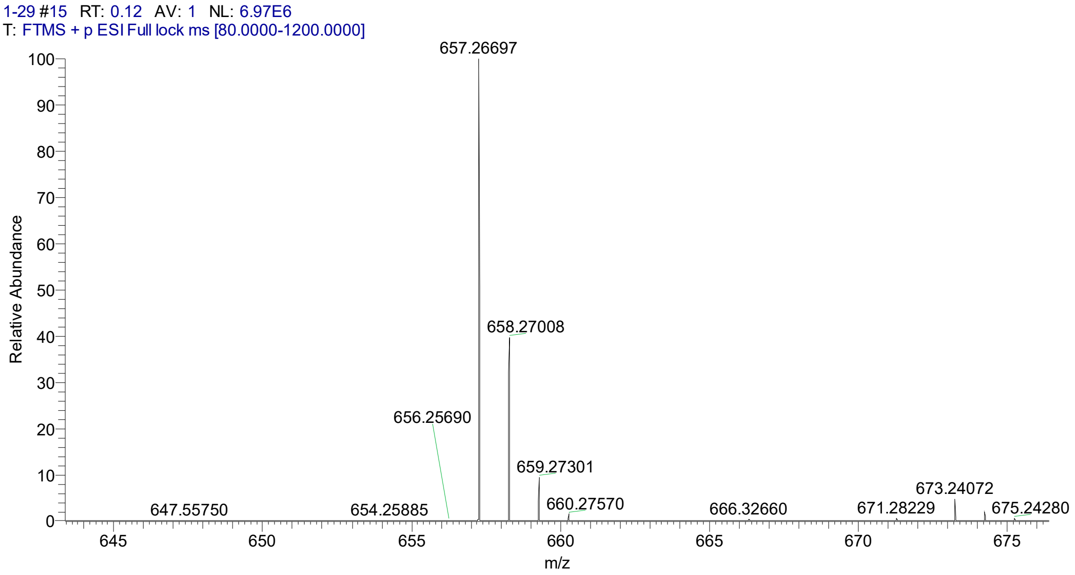

Figure S14. The HRESIMS spectrum of **2**.
